# The NADPH oxidase NOX2 as a novel biomarker for suicidality: evidence from human *post mortem* brain samples

**DOI:** 10.1038/tp.2016.76

**Published:** 2016-05-17

**Authors:** S Schiavone, M Neri, E Mhillaj, M G Morgese, S Cantatore, M Bove, I Riezzo, P Tucci, C Pomara, E Turillazzi, V Cuomo, L Trabace

**Affiliations:** 1 Department of Clinical and Experimental Medicine, University of Foggia, Foggia, Italy; 2 Department of Physiology and Pharmacology, 'Sapienza' University of Rome, Rome, Italy

## Abstract

Recent evidence points towards a role of oxidative stress in suicidality. However, few studies were carried out on the sources of reactive oxygen species (ROS) in subjects with suicidal behaviour. We have previously demonstrated that the NADPH oxidase NOX2-derived oxidative stress has a major role in the development of neuropathological alterations observed in an animal model of psychosis. Here, we investigated the possible increase in NOX2 in *post mortem* brain samples of subjects who died by asphyctic suicide (AS) compared with controls (CTRL) and subjects who died by non-suicidal asphyxia (NSA). We found that NOX2 expression was significantly higher in the cortex of AS subjects than in the other two experimental groups. NOX2 immunostaining was mainly detected in GABAergic neurons, with a minor presence of NOX2-positive-stained cells in glutamatergic and dopaminergic neurons, as well as astrocytes and microglia. A sustained increase in the expression of 8-hydroxy-2'-deoxyguanosine, an indirect marker of oxidative stress, was also detected in the cortex of AS subjects, compared with CTRL and NSA subjects. A significant elevation in cortical interleukin-6 immunoreactivity in AS subjects suggested an involvement of cytokine-associated molecular pathways in NOX2 elevations. Our results suggest that the increase in NOX2-derived oxidative stress in the brain might be involved in the neuropathological pathways leading to suicidal behaviour. These results may open innovative insights in the identification of new pathogenetic and necroscopic biomarkers, predictive for suicidality and potentially useful for suicide prevention.

## Introduction

Psychiatric disorders, such as depression, bipolar disorders and psychosis, are associated with high risk of suicide.^1^ In modern societies, suicide represents a major public health problem and, in particular, suicide by asphyxia appears to be the most common suicidal modality in European patients.^2, 3, 4^ Thus, violent asphyxia remains a common cause of death in the practice of forensic pathologists.^5^ However, current knowledge on the neurobiological factors associated with suicide risk is still poor. A number of reports pointed out that affective illness and psychosocial distress could represent major risk factors for suicidal behaviour, and several studies have described biochemical changes in *post mortem* brain samples of suicidal subjects.^6^ Although a large amount of data focus on forensic and legal aspects of suicide, few investigations have been conducted to identify molecular pathways leading to suicidality. Increasing evidence shows a possible contribution of brain oxidative stress and increased reactive oxygen species (ROS) production in the central nervous system (CNS) in the development of suicidal behaviour.^7^ Possible sources of ROS include the NADPH oxidase NOX2 enzyme, which is a protein that transfers electrons across biological membranes to produce superoxide. This enzyme is constitutively expressed in the CNS and is a major generator of ROS in several pathological conditions, from psychiatric to neurodegenerative diseases.^8^ In particular, we recently demonstrated that early NOX2 increase in specific brain areas contributes to the development of neuropathological alterations observed in non-pharmacologic and pharmacologic rodent models of psychosis, such as the social isolation and the ketamine model, respectively.^9, 10, 11^

Multiple studies have shown that high circulating interleukin-6 (IL-6) is positively associated with enhanced risk of disease and disability development.^12^ In particular, cerebral IL-6 expression has been shown to mediate alterations of CNS functioning, including modulation of glutamatergic neurotransmission.^13^ In the ketamine rodent model of psychosis, administration of this dissociative anaesthetic drug to young mice increased expression of IL-6 in the brain, as well as of the NADPH oxidase NOX2 (ref. 14).

Collections of *post mortem* brain samples and brain banking represent very useful tools for improving our understanding of molecular mechanisms associated with neuropsychiatric diseases, for identifying new diagnostic targets and for developing new therapeutic and prevention strategies.^15^

Thus, the main aim of this study was to evaluate the possible role of NOX2-derived oxidative stress in suicidal behaviour. To this purpose, we analysed a total of 42 human autoptic brain specimens divided into three different experimental groups: subjects who died by asphyctic suicide (*n*=26; AS), subjects who died by non-suicidal asphyxia (*n*=6; NSA) and subjects who died by other causes of death (nor suicide or NSA) as controls (CTRL; *n*=10). *Post mortem* brain samples of these subjects were analysed for expression of NOX2 and other markers of oxidative stress with immunohistochemistry. NOX2 expression in cellular brain subpopulations (neurons, microglia and astrocytes) was also evaluated. Finally, the possible involvement of IL-6-associated pathways in NOX2 elevations was investigated.

## Materials and methods

### *Post mortem* sample recruitment

Autopsy records of autopsies performed at the Section of Legal Medicine, Department of Clinical and Experimental Medicine, University of Foggia, Italy, from 2001 to 2014 were evaluated. A total number of 26 *post mortem* brain samples of AS subjects (17 cases of hanging and 9 cases of strangulation), 10 *post mortem* brain samples of CTRL subjects (died following road accidents without brain injury or cardiac sudden deaths) and 6 *post mortem* brain samples of NSA subjects (1 case of smothering, 1 case of choking, 1 case of positional asphyxia and 3 cases of traumatic asphyxia by chest compression) were recruited. Exclusion criteria included deaths following accidental poisoning, organophosphate-induced asphyxia, presence of severe infectious diseases (such as AIDS) and recreational abuse of ketamine. Toxicological analyses for the most common drugs of abuse (cocaine, heroin, amphetamine, methadone and cannabinoids) were negative for all subjects included in the study.

### Sociodemographic distribution

Sociodemographic distribution of subjects included in the study is reported in Table 1.

### Technical details

For all subjects included in the study, autopsies were performed between 24 and 48 h after death. To avoid the progression of transformative phenomena, bodies were kept in cold storage room (−5 °C) until the performance of autopsy.

### Histological and immunohistochemical study

A routine microscopic histopathological analysis was performed on brain samples, collected during autopsies. In particular, specimens derived from the frontal cortex were processed and stained with haematoxylin–eosin.

Expression of NOX2 enzyme and markers of oxidative stress was evaluated using anti-Nox 2 (Santa Cruz Biotechnology, Santa Cruz, CA, USA) and anti-8-hydroxy-2'-deoxyguanosine (8-OHdG) antibodies (JaICA, Shizuoka, Japan). Neuroinflammation was investigated using anti-IL-6 (Santa Cruz Biotechnology), anti-TNF (tumour necrosis factor)-α (Santa Cruz Biotechnology), anti-IL-10 (Peprotec, London, UK) and anti-IL-1 beta (Santa Cruz Biotechnology) antibodies.

Briefly, 4-μm-thick paraffin sections mounted on slides covered with 3-aminopropyltriethoxysilane (Fluka, Buchs, Switzerland) were used. Proteolytic enzyme (Dako, Copenhagen, Denmark) pretreatment at 20 °C (for anti-IL-6 and anti-IL-10) or microwave pretreatment in 0.25 m EDTA buffer (for anti-IL1β) and in 0.1 m citric acid buffer (for anti-TNFα, anti-NOX2 and anti-8-OHdG) were performed to facilitate antigen retrieval and to increase membrane permeability. Dilutions of primary antibodies were as follows: 1:10 for 8-OHdG, 1:50 for NOX2, 1:600 for TNFα, 1:4000 for IL-1 beta and 1:2000 for IL-6 and IL-10. Incubation time was 120 min at room temperature. The used detection system consisted of a refined avidin–biotin technique in which a biotinylated secondary antibody reacted with several peroxidase-conjugated streptavidin molecules. The peroxidase–avidin/biotin complex was visualised using DAB (3,3-diaminobenzidinetetrahydrochloride hydrate) with a brown reaction. Sections were finally counterstained with Mayer's haematoxylin.

To identify the cellular subtype involved in NOX2 increase, double immunohistochemistry was performed using various peroxidase substrates with different colours: Vector NovaRED (red), Vector VIP (purple), Vector SG (blue/grey) and DAB (brown; Vector, Burlingame, CA, USA). Brain tissue pretreatment and NOX2 detection were conducted as previously described,^9, 10, 11^ and the peroxidase–avidin/biotin complex was visualised using Vector SG with a blue/grey reaction. Slices were incubated with the following primary antibodies: Neun (Abcam, Cambridge, UK) 1:1000 ratio, glial fibrillary acidic protein (GFAP) (Santa Cruz Biotechnology) 1:300 ratio, MAC387 (Santa Cruz Biotechnology) 1:200 ratio, GAD67 (Abcam) 1:2000 ratio, VGLUT1 (Abcam) 1:500 ratio and DT1 (Abcam) 1:100 ratio. After the incubation with the detection system, the peroxidase–avidin/biotin complex was visualised using Vector NovaRED (red reaction) for MAC387 and GAD67, Vector VIP (purple reaction) for Neun and GFAP, and DAB (brown reaction) for DT1 and VGLUT1. Sections were counterstained with methyl green, dehydrated, coverslipped and observed in a Leica DM6000 optical microscope (Leica, Cambridge, UK).

Quantification of NOX2, 8-OHdG, IL-6, TNFα, IL-10 and IL-1 beta-positive-stained cells was performed using the ImageJ software (imagej.nih.gov/ij/) and expressed as number of positive stained cells/analysed area.

### Statistical analysis

Data were analysed using the GraphPad Prism 5 software for Windows (La Jolla, CA, USA). Data were checked for normality. Parametric data were analysed by one-way analysis of variance (ANOVA), followed by Tukey's *post hoc* test. Non-parametric data were analysed by Kruskal–Wallis followed by Dunn's multiple comparisons test. For all tests, a *P*-value<0.05 was considered statistically significant. Results are expressed as means±s.e.m. in graphs. s.d.'s are indicated in the Results section.

## Results

### Increase in NOX2 and 8-OHdG immunostaining in the cortex of AS subjects

**Figure 1:**
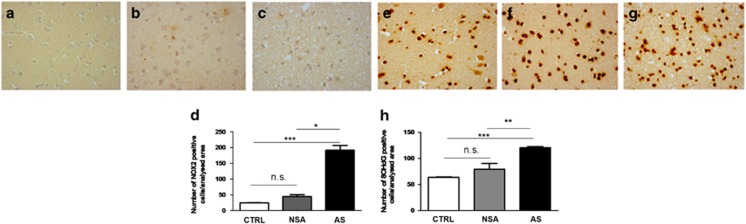
Increase of NOX2 and 8-hydroxy-2'-deoxyguanosine (8-OHdG) immunostaining in the cortex of suicidal subjects. (**a**–**c**) Representative images of NOX2 immunostaining in the cortex of controls (CTRL; **a**, *n*=10), subjects who died by non-suicidal asphyxia (NSA; **b**, *n*=6) and by asphyctic suicide (AS; **c**, *n*=26). (**d**) Quantification of NOX2-positive-stained cells in the cortex of CTRL (*n*=10), subjects who died by NSA (*n*=6) and by AS (*n*=26). Kruskal–Wallis, followed by Dunn's multiple comparisons test, ***P<0.001; *P<0.05; n.s.=not significant. (**e**–**g**) Representative images of 8-OHdG immunostaining in the cortex of CTRL (**e**, *n*=10), subjects who died by NSA (**f**, *n*=6) and by AS (**g**, *n*=26). (**h**) Quantification of 8-OHdG-positive-stained cells in the cortex of CTRL (*n*=10), subjects who died by NSA (*n*=6) and by AS (*n*=26). Kruskal–Wallis, followed by Dunn's multiple comparisons test, ***P<0.001; **P<0.01; n.s.=not significant.

In order to investigate whether NOX2-derived oxidative stress might be involved in deaths by suicide, we performed immunohistochemical analysis for NOX2, focusing on the cortex of AS subjects, compared with CTRL and NAS subjects. NOX2 immunostaining was under detection levels in CTRL subjects. Whereas the presence of only few NOX2-positive-stained cells was observed in NSA subjects, a significant increase in the number of NOX2-immunoreactive cells was detected in AS subjects (Figures 1a–d, Kruskal–Wallis, followed by Dunn's multiple comparisons test, ***P<0.001; *P<0.05, s.d. CTRL=2.675; s.d. NSA=15.12; s.d. AS=67.93). Contingency analysis revealed that the increase in NOX2 expression observed in the cortex of AS subjects was not correlated to gender (Supplementary Figure 1A; *X*^2^, degree of freedom=0.62501; *P*=0.4292) and age group (Supplementary Figure 1B; *X*^2^, degree of freedom=3.0392; *P*=0.2188). Importantly, a concomitant significant elevation of 8-OHdG immunoreactivity was detected in the cortex of AS subjects, with respect to CTRL and NSA subjects (Figures 1e–h, Kruskal–Wallis followed by Dunn's multiple comparisons test, ***P<0.001; **P<0.01, s.d. CTRL=3.502; s.d. NSA=27.03; s.d. AS=8. 682).

### Increase in NOX2 immunostaining in GABAergic cortical neurons of AS subjects

**Figure 2:**
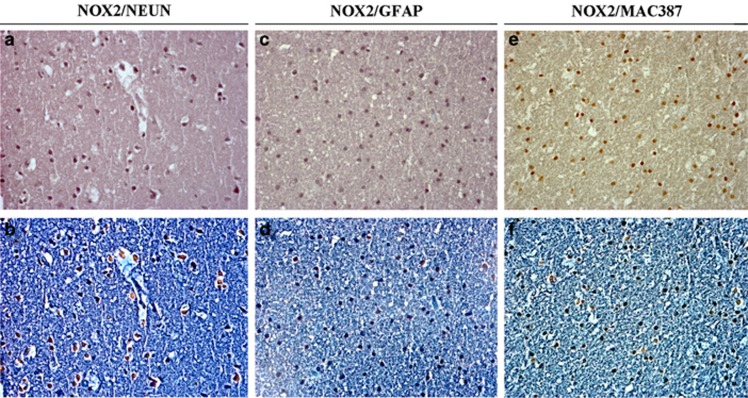
NOX2 immunostaining in neurons of suicidal subjects. (**a**, **b**) Representative images of double immunostaining for NOX2/Neun in the cortex of suicidal subjects (*n*=26) in bright field (**a**) and contrast phase (**b**). (**c,d**). Representative images of double immunostaining for NOX2/glial fibrillary acidic protein (GFAP) in the cortex of suicidal subjects (*n*=26) in bright field (**c**) and contrast phase (**d**). (**e**, **f**) Representative images of double immunostaining for NOX2/MAC387 in the cortex of suicidal subjects (*n*=26) in bright field (**e**) and contrast phase (**f**).

To evaluate which brain cellular subpopulation was involved in NOX2 expression increase, we performed double immunohistochemistry for NOX2 and Neun, an immunohistochemical marker used for neuronal identification, NOX2 and GFAP, an immunohistochemical marker used to detect astrocytes, and NOX2 and MAC387, used to identify microglia, in the cortex of AS subjects. We found that NOX2 was mainly expressed in Neun-positive-stained cells of AS subjects (Figures 2a and b), whereas a minor percentage of NOX2/GFAP and NOX2/MAC387 co-staining was detected in astrocytes (Figures 2c and d) and microglia, respectively (Figures 2e and f). Thus, NOX2 was mainly increased in cortical neurons of AS subjects.

**Figure 3:**
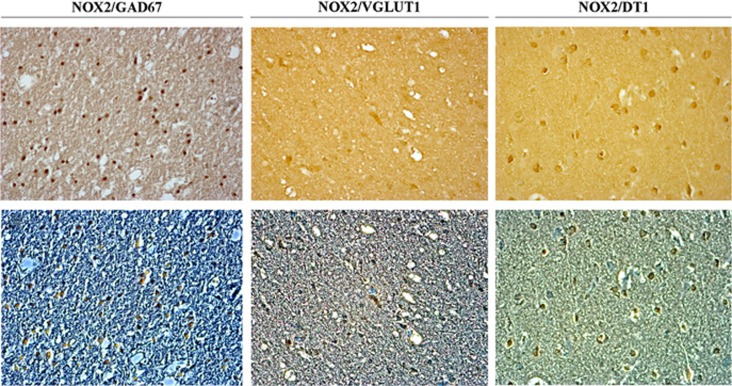
NOX2 immunostaining in cortical GABAergic neurons of suicidal subjects. (**a,** **b**) Representative images of double immunostaining for NOX2/GAD67 in the cortex of suicidal subjects (*n*=26) in bright field (**a**) and contrast phase (**b**). (**c**, **d**). Representative images of double immunostaining for NOX2/VGLUT1 in the cortex of a suicidal subjects (*n*=26) in bright field (**c**) and contrast phase (**d**). (**e, f**) Representative images of double immunostaining for NOX2/DT1 in the cortex of a suicidal subjects (*n*=26) in bright field (**e**) and contrast phase (**f**).

To further investigate which neuronal subtype was specifically involved in NOX2-increased expression, we performed double immunohistochemistry for NOX2 and GAD67, an immunohistochemical marker of GABAergic neurons, NOX2 and VGLUT1, an immunohistochemical marker of glutamatergic neurons, and NOX2 and DT1, an immunohistochemical marker of dopaminergic neurons. Results showed the presence of NOX2 and GAD67 co-staining in the cortex of AS subjects (Figures 3a and b). A very weak co-staining for NOX2 and VGLUT1 (Figures 3c and d), as well as for NOX2 and DT1 was detected (Figures 3e and f). Thus, results indicated that increase in NOX2 expression mainly involves cortical GABAergic neurons of AS subjects.

### Increase in NOX2 in AS subjects is associated with IL-6 increase

**Figure 4:**
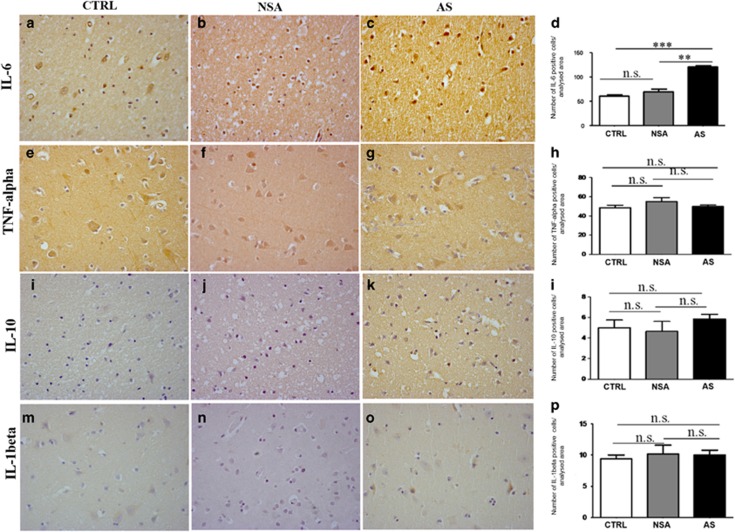
Increase of NOX2 in suicidal subjects is associated with interleukin-6 (IL-6). (**a**–**c**) Representative images of IL-6 immunostaining in the cortex of controls (CTRL; **a**, *n*=10), subjects who died by non-suicidal asphyxia (NSA; **b**, *n*=6) and by asphyctic suicide (AS; **c**, *n*=26). (**d**) Quantification of IL-6-positive-stained cells in the cortex of CTRL (*n*=10), subjects who died by NSA (*n*=6) and by AS (*n*=26). Kruskal–Wallis followed by Dunn's multiple comparisons test ***P<0.001; **P<0.01; n.s.=not significant. (**e**–**g**) Representative images of tumour necrosis factor alpha (TNFα) immunostaining in the cortex of CTRL (**e**, *n*=10), subjects who died by NSA (**f**, *n*=6) and by AS (**g**, *n*=26). (**h**) Quantification of TNFα-positive-stained cells in the cortex of CTRL (*n*=10), subjects who died by NSA (*n*=6) and by AS (*n*=26). One-way analysis of variance (ANOVA) followed by Tukey's *post hoc* test, F=1.372, n.s.=not significant. (**i**–**k**) Representative images of IL-10 immunostaining in the cortex of CTRL (**i**, *n*=10) and in subjects who died by NSA (**j**, *n*=6) and by AS (**k**, *n*=26). (**l**) Quantification of IL-10-positive-stained cells in the cortex of CTRL (*n*=10), subjects who died by NSA (*n*=6) and by AS (*n*=26). One-way ANOVA followed by Tukey's *post hoc* test, F=0.9291, n.s.=not significant. (**m**–**o**) Representative images of IL-1 beta immunostaining in the cortex of CTRL (**m**, *n*=10), subjects who died by NSA (**n**, *n*=6) and by AS (**o**, *n*=26). (**p**) Quantification of IL-1 beta-positive-stained cells in the cortex of CTRL (*n*=10), subjects who died by NSA (*n*=6) and by AS (*n*=26). One-way ANOVA followed by Tukey's *post hoc* test, F=0.1471, n.s.= not significant.

To identify molecular mechanisms leading to the increase in NOX2 in the cortex of AS subjects, we investigated the possible involvement of neuroinflammation and, in particular, alterations of specific cytokines, such as IL-6, TNFα, IL-10 and IL-1 beta. Whereas immunohistochemical analysis revealed the presence of a weak IL-6 immunoreactivity in CTRL and NAS subjects, the number of IL-6-positive-stained cells was dramatically increased in AS subjects (Figures 4a–d, Kruskal–Wallis followed by Dunn's multiple comparisons test ***P<0.001; **P<0.01, s.d. CTRL=8.208; s.d. NSA=13.87; s.d. AS=8.957). No significant differences in the number of TNFα (Figures 4e–h, one-way ANOVA followed by Tukey's *post hoc* test, F=1.372, s.d. CTRL=8.959; s.d. NSA=10.02; s.d. AS=6.945), IL-10 (Figures 4i–l, one-way ANOVA followed by Tukey's *post hoc* test, F=0.929; s.d. CTRL=2.404; s.d. NSA=2.338; s.d. AS=1.975) and IL-1 beta (Figures 4m–p, one-way ANOVA followed by Tukey's *post hoc* test, F=0.1471; s.d. CTRL=1.897; s.d. NSA=3.488; s.d. AS=3.659)-positive-stained cells were detected between CTRL, NAS and AS subjects.

## Discussion

In this study, we evaluated a possible increase in NOX2-derived oxidative stress in human *post mortem* brain samples of AS subjects. We found that NOX2 expression was significantly increased in the cortex of AS subjects with respect to CTRL and NSA subjects. NOX2 elevation was associated to a dramatic increase in another marker of oxidative stress, the 8-OHdG.

Our results are in line with recent findings reporting oxidative stress and lowered total antioxidant status in depressed subjects, with a history of suicide attempts. Indeed, in a recent work, Vargas *et al.*^7^ demonstrated an increased level of nitric oxide metabolites, lipid hydroperoxides, malondialdehyde, as well as advanced oxidation protein products and reduced plasmatic antioxidant potential, in blood specimens of individuals with a positive anamnesis for major depression and who attempted suicide. In this context, nitric oxide-derived oxidative stress, related to a polymorphism of the *nitric oxide synthase 3* gene, has been recently shown to be involved in the development of violent suicidal behaviour in subjects suffering from bipolar disorders.^16^ In addition, a genetic polymorphism in *Glutathione-S-transferase*, leading to decreased antioxidant capacities, has been recently associated with anxiety, mood disorders and increased risk of suicide attempts.^17^ Some studies have also pointed towards a possible effect of antioxidant compounds, such as selenium,^18^ vitamin E,^19^ vitamin D^20^ and vitamin A,^21^ in preventing or decreasing suicidal behaviour, especially in depressed patients. Interestingly, treatment with the glutathione precursor *N*-acetylcysteine has been proposed for the prevention of suicidal behaviour in psychiatric patients.^22^ With respect to the observed NOX2 increase, a limitation of this study might be represented by the small sample size of the NSA subjects. However, despite the small sample size of this group, the low variability found within the NSA subjects encouraged us to consider this result as a non-spurious finding. Moreover, given the features of this experimental group, it was particularly difficult to increase the sample size.

The role of the NADPH oxidase NOX2 in human pathologies has been well documented, ranging from chronic granulomatous disease,^23, 24, 25^ acute myocardial infarction^26^ to neurodegenerative disorders.^8^ However, concerning the role of NOX2 in the pathogenesis of psychiatric disorders, all available data have been obtained in animal or cellular models. We have previously demonstrated that an early NOX2 increase in specific brain areas contributes to the development of neuropathological alterations in non-pharmacologic and pharmacologic rodent models of psychosis, such as the social isolation and ketamine models.^9, 10, 11^ However, although animal models of psychosis are undoubtedly useful tools to reproduce some aspects of the human psychosis, such as neurochemical and behavioural alterations reminiscent of those observed in psychotic patients, they still remain far from the complexity of the human pathology, especially in regard to psychopathological, emotional and social aspects. To the best of our knowledge, no evidence of the involvement of NOX2 in human psychiatric diseases is actually available. Thus, our results might be considered specific for suicidality, being, therefore, the first evidence of NOX2 increase in suicidal behaviour in humans.

A crucial finding of this study is that increased levels of NOX2 expression were mainly observed in cortical GABAergic neurons. Interestingly, this neuronal subpopulation has been shown to be exposed to several pathological alterations, such as loss of fast-spiking parvalbumin-positive interneurons in animal models of psychiatric disorders^14, 27^ or changes in the density of calcium-binding protein immunoreactivity in subjects suffering from schizophrenia and bipolar disorders.^28, 29^ An altered expression of genes involved in GABAergic neurotransmission has also been identified in the ventral prefrontal cortex of suicidal subjects with or without major depression.^30^ In this line, Poulter *et al.*^31^ reported gene-specific aberrations in DNA methylation of GABA-A receptor α1 subunit promoter region, within the frontopolar cortex, in the brains of suicidal/major depressed subjects. Interestingly, GABA concentration in cerebrospinal fluid appears to positively correlate with impulsiveness and history of suicidal behaviour in personality disordered subjects,^32^ although disinhibition is observed only in a minority of cases. This may be consistent with observations that high doses of benzodiazepines can lead to ‘behavioural disinhibition', including suicide attempts, in human subjects.^33^

**Figure 5:**
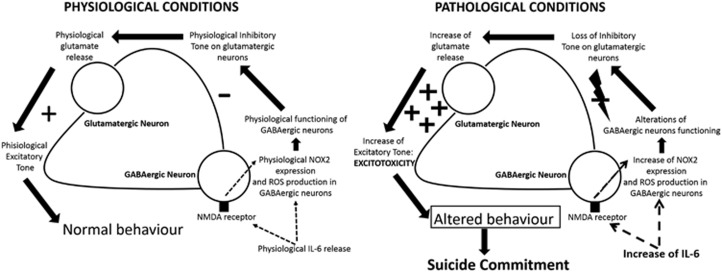
Hypothetical molecular mechanism linking interleukin-6 (IL-6) increase to NOX2 elevation in suicidal behaviour. Under physiological conditions, basal IL-6 release might be involved in the maintenance of physiological NOX2 expression in GABAergic neuron levels and ROS production by interacting with the N-methyl-D-aspartate (NMDA) receptor placed on GABAergic neurons. This might permit the maintenance of the physiological inhibitory tone of GABAergic neurons on glutamatergic ones to assure the release of basal glutamate levels and the physiological excitatory tone, necessary to preserve a normal behaviour. Under pathological conditions, the increase in IL-6 levels might mediate NOX2 elevations as well as NOX2-derived ROS production in GABAergic neurons, leading to an altered functioning of this neuronal population, loss of inhibitory tone and, finally, increased glutamate release and excitotoxicity associated to altered behaviour and death by suicide.

Another crucial aspect of this study is the significant increase in IL-6 expression in the cortex of AS subjects with respect to CTRL and NAS subjects. Our data are in line with recent findings showing IL-6 elevations in the cerebrospinal fluid of depressed suicide attempters and a pathological link between increased levels of this cytokine and the severity of suicide ideation and depressive symptoms.^34^ Importantly, IL-6 has been shown to be necessary and sufficient for NOX2 increase in the ketamine model of schizophrenia,^14^ as well as the degeneration of forebrain GABAergic interneurons through activation of the neuronal NADPH oxidase.^14, 35^ A possible molecular mechanism linking NOX2 increase in GABAergic neurons, IL-6 elevations and suicidal behaviour is represented in Figure 5 and described in details in its legend. Although still speculative, this hypothetical mechanism is supported by studies demonstrating increased glutamate levels^36^ and altered glutamate signalling^37^ in brains of suicidal patients with mood disorders. In addition, microarray analysis in the cerebral cortex of individuals who had suffered from major depressive disorder and died by suicide demonstrated significant downregulation of the key members of the glutamate/neutral amino-acid transporter protein family.^38^ Other evidence supporting this hypothesis deals with the fact that neurotoxic effects of IL-6 appear to be mediated by its molecular interaction with the N-methyl-D-aspartate receptor.^39^ A possible contribution of other cellular populations, such as microglia, in NOX2-derived ROS increase cannot be ruled out. Thus, we found a minor percentage of NOX2/MAC387 co-staining in the cortex of AS subjects. This result is in agreement with a study demonstrating that microglia is directly involved in the development of pre-suicidal stress through the release of different factors, which, in turn, appear to act on noradrenergic and serotonergic neurotransmission.^40^ Furthermore, in a recent study of Schnieder *et al.*, Authors demonstrated greater density of ionised calcium-binding adapter molecule 1-immunoreactive cells in the dorsal prefrontal white matter of suicidal subjects.^41^

A possible involvement of other sources of oxidative stress in the development of suicidal behaviour, such as mitochondria, must be considered. Indeed, prevention of mitochondria-derived ROS production by consumption of specific nutrients such as ω-3 fatty acids, vitamin C, zinc, members of the vitamin B family (Vitamin B12 and folic acid) and magnesium may help to prevent the onset of mood disorders and suicidal behaviours in vulnerable individuals.^42^

Subjects included in this study died by AS. Thus, a possible question could arise: might this suicidal modality have a direct impact on NADPH oxidase expression and NOX2-derived oxidative stress production? To exclude the possibility that NOX2 increase might be due to asphyxia and not to suicidal behaviour, we also included, in our study, NAS subjects. Importantly, although a link between asphyxia and the production of ROS, such as peroxynitrite, nitric oxide and nitrogen dioxide, has been demonstrated in rodent models of perinatal and neonatal asphyxia,^43^ as well as under this pathological condition in humans,^44^ we did not detect any statistical differences between NAS subjects and CTRL. This strongly argues in favour of a specific role of suicidal behaviour in determining NOX2 and oxidative stress-marker elevation. The lack of NOX2 elevation in the group of NAS subjects is also in line with evidence demonstrating that hypoxia leads to the production of nitric oxide and superoxide, essentially through the increased expression and activation of cytoplasmic,^45^ as well as mitochondrial,^46^ nitric oxide synthases and through the generation of superoxide anions, essentially by mitochondria.^47^ However, a minor impact of asphyxia on NADPH oxidase expression and activity cannot be totally excluded. Indeed, asphyxia has been shown to induce an accumulation of neutrophils in asphyctic organs, in particular the brain, associated with a production of ROS via NADPH oxidase and of hypochlorous acid via myeloperoxidase.^48^

Another important aspect that certainly requires attention is the possible impact of medication on the observed NOX2 increase. Thus, within AS subjects, one was medicated with citalopram, haloperidol and promazine. To this regard, literature reported some data on the possible link existing between peripheral side effects of this medication (such as erectile dysfunctions, pulmonary hypertension, platelet hyperaggregability and agranulocytosis) and increased NADPH oxidase-derived ROS production.^49, 50, 51^ To the best of our knowledge, no data are actually available describing a possible increase in NOX-derived brain oxidative stress because of this medication. Thus, although it has been shown that haloperidol induces oxidative stress in the rat brain, this has been mainly attributed to mitochondrial superoxide generation and production of nitric oxide by nitric oxide synthase enzymes.^52, 53, 54, 55^ However, a possible effect of these pharmacological compounds on the observed increased in NOX2 cannot be totally excluded.

Measuring NOX-enzyme activity is a crucial step in understanding their physiological and pathological roles. Thus, a limitation of this study is certainly represented by the lack of data concerning NOX2 activity in these human brain samples. It appears to us particularly important to underline that efficient and reliable methods to directly measure NOX activity actually represent the major breakthrough in the field of NOX and oxidative stress.^56^ Thus, the most commonly used methods could lead to artefacts and misinterpretation. Novel methods for *in vivo* measurement of NOX activity are emerging, and they appear to be specific for the detection of oxidants in the brain.^57^

A limiting step in suicide prediction and prevention is the lack of objective and reliable predictors.^58^ Interestingly, in this study, we suggest the NADPH oxidase NOX2 as a novel biomarker for suicide and suicidal behaviour. In this context, it seems important to highlight that biomarkers for suicidality have been studied using principally blood samples of subjects with major mood disorders or psychosis,^59^ two groups of psychiatric disorders in which the role of NOX2-derived oxidative stress has been recently well documented.^9, 10, 11, 60, 61^ Recently, Guintivano *et al.* also performed a genome-wide DNA methylation profiling of three independent data sets of post-mortem brains of suicidal subjects, identifying signs of increased methylation in one particular gene, *SKA2*.^62^ Intriguingly, the protein derived from this gene mainly acts via a ‘redox-sensitive' pathway,^63^ being also implicated in molecular mechanisms of protection against ROS-derived neurotoxicity.^64^

Together with the possible use of NOX2 as a novel biomarker for prediction and prevention of suicide, our study might be also considered in the light of other possible clinical utilities. Thus, a pathological link between increased oxidative stress in suicidal subjects and alterations of brain morphology have been reported^65^ and, interestingly, these brain alterations have been identified with specific brain imaging techniques.^66^

## Conclusions

The neurobiology of suicide is complex and remains still not completely understood. Despite accumulating data exploring the interaction between genetic, environmental, microstructural and developmental factors involved in the suicide phenomenon, no formal consensus exists in associating a neurobiological mechanism to suicide. Post-mortem studies may be useful in characterizing the suicide profile as suicide completers and suicide attempters are only partially joined by common aetiological and neurobiological mechanisms, and suicide completers are considered a more homogeneous group than suicide attempters.^67^ Defining some potential biomarkers for suicidality could help in determining modalities and circumstances of death that, in turn, may improve our understanding of suicidal behaviour. Our results strongly support the hypothesis that pharmacological targeting of NOX2 might be crucial for the treatment and the reversion of several behavioural alterations, such as suicide.

## Figures and Tables

**Table 1:** Sociodemographic distribution

*Characteristics*	*Controls (*n*=10)*	*Non-suicidal asphyxia (*n*=6)*	*Asphyctic suicide (*n*=26)*
*Gender*			
Male	8	2	21
Female	2	4	5
			
*Age group*			
18–40	3	2	18
40–70	6	1	4
>70	1	3	4
			
*Race*			
Caucasian	10	6	26
Black	0	0	0
Yellow	0	0	0
Mixed	0	0	0
			
*Marital status*			
Single	2	0	5
Stable relationship	7	4	9
Divorced/separated	1	0	3
Widow	0	1	1
Not known	0	1	8
			
*Psychiatric anamnesis*			
Negative/not known	10	6	18
Positive	0	0	8
			1 Delirium of persecution
			1 Behaviour disorders
			3 Aspecific psychiatric disorder
			3 Alcohol addiction
			
*Other stressful conditions*			
Negative/not known	10	6	9
Positive	0	0	17
			4 Detention
			1 Pregnancy
			6 Recent outage of a romantic relationship
			2 Neoplastic diseases
			2 Recent lost of job
			2 Homicide-suicide
			
*Medication*			
Not known	10	6	25
Psychiatric medication	0	0	1
			Citalopram 20 mg/die
			Haloperidol 30 mg/die
			Promazine 50 mg according to needs

## Supplementary Material

Suppmentary Information
