# Supplementary figures and images for: The NADPH oxidase NOX2 as a novel biomarker for suicidality: evidence from human post mortem brain samples
Source: Transl Psychiatry. 2016 May 17;6(5):e813–. doi: 10.1038/tp.2016.76 (PMC5070044; doi:10.1038/tp.2016.76)

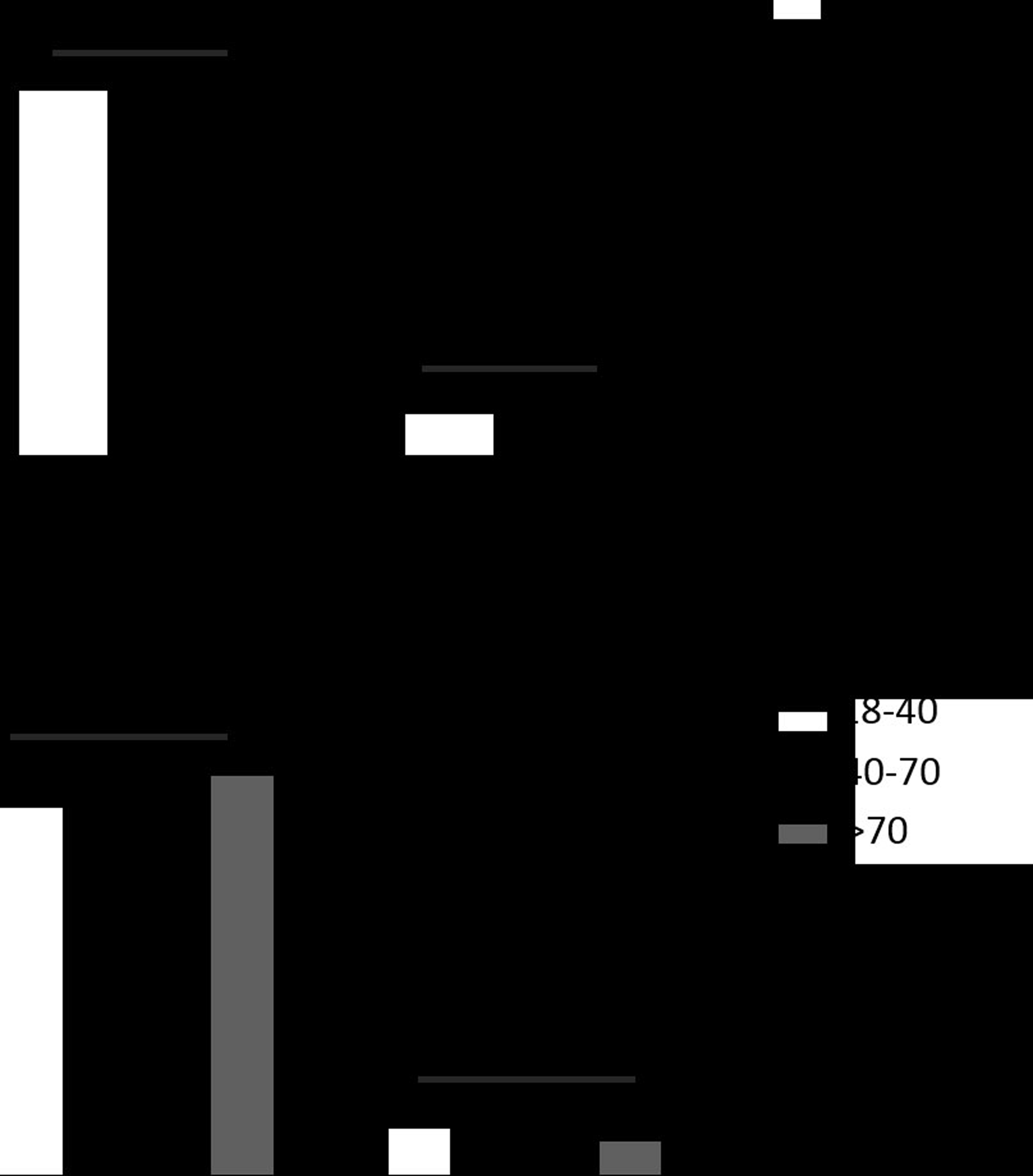

Supplement: Suppmentary Information [file tp201676x1.tif]
