# Supplementary material for: The NADPH oxidase NOX2 as a novel biomarker for suicidality: evidence from human post mortem brain samples
Source: Transl Psychiatry. 2016 May 17;6(5):e813–. doi: 10.1038/tp.2016.76 (PMC5070044; doi:10.1038/tp.2016.76)
Supplement: Supplementary Figure 1 Legend [file tp201676x2.doc]

**Supplementary Fig. 1 Increase in NOX2 expression in the cortex of AS subjects was not correlated to gender or age group**

1. Contingency analysis for the number of cortical NOX2 positive cells/ analysed area and gender (male and female). Chi square test X2**,** df 0.6250,1; p=0,4292; n.s.= not significant
2. Contingency analysis for the number of cortical NOX2 positive cells/ analysed area and age group (18-40, 40-70 and >70). Chi square test X2**,** df 3.039,2; p=0,2188; n.s.= not significant
